# Can volumetric modulated arc radiation therapy reduce organ at risk dose in stage 4 sinonasal tumors in dogs treated with boost irradiation?

**DOI:** 10.1371/journal.pone.0259112

**Published:** 2021-10-29

**Authors:** Valeria Meier, Felicitas Czichon, Linda Walsh, Carla Rohrer Bley

**Affiliations:** 1 Vetsuisse Faculty, Department for Small Animals, Division of Radiation Oncology, University of Zurich, Zurich, Switzerland; 2 Department of Physics, University of Zurich, Zurich, Switzerland; Colorado State University, UNITED STATES

## Abstract

Intensity modulated radiation therapy (IMRT) introduced marked changes to cancer treatment in animals by reducing dose to organs at risk (OAR). As the next technological step, volumetric modulated arc therapy (VMAT) has advantages (increased degrees-of-freedom, faster delivery) compared to fixed-field IMRT. Our objective was to investigate a possible advantage of VMAT over IMRT in terms of lower OAR doses in advanced-disease sinonasal tumors in dogs treated with simultaneously-integrated boost radiotherapy. A retrospective, analytical, observational study design was applied using 10 pre-existing computed tomography datasets on dogs with stage 4 sinonasal tumors. Each dataset was planned with both, 5-field IMRT and 2 arc VMAT with 10x4.83 Gy to the gross tumor volume and 10x4.2 Gy to the planning target volume. Adequate target dose coverage and normal tissue complication probability of brain ≤5% was required. Dose constraints aspired to were D60 <15 Gy for eyes, D2 <35.4 Gy for corneae, and Dmean <20 Gy for lacrimal glands. OAR dose was statistically significantly higher in IMRT plans than in VMAT plans. Median eye D60% was 18.5 Gy (interquartile range (IQR) 17.5) versus 16.1 Gy (IQR 7.4) (p = 0.007), median lacrimal gland dose 21.8 Gy (IQR 20.5) versus 18.6 Gy (IQR 7.0) (p = 0.013), and median cornea D2% 45.5 Gy (IQR 6.8) versus 39.9 Gy (IQR 10.0) (p<0.005) for IMRT versus VMAT plans, respectively. Constraints were met in 21/40 eyes, 7/40 corneae, and 24/40 lacrimal glands. Median delivery time was significantly longer for IMRT plans than for VMAT plans (p<0.01). Based on these results, VMAT plans were found to be superior in sparing doses to eyes, lacrimal glands, corneae. However, not all ocular OAR constraints could be met while ensuring adequate dose coverage and restricting brain toxicity risk for both planning techniques.

## Introduction

Intensity modulated radiation therapy (IMRT) has introduced marked changes to the treatment of malignant tumors in animals. In terms of tumor control, IMRT does not by itself produce better results than appropriate 3-dimensional conformal radiation therapy (3D-CRT) in canine brain tumors [1]. The unprecedented accuracy of dose delivery in IMRT, however, often massively reduces toxicity in organs at risk (OAR) without compromising dose coverage to the planning target volume (PTV) [2–5]. Hence, as a perspective, IMRT allows for changes in treatment fractionation, absorbed-dose escalation or boost therapy, features with a strong potential to increase tumor control [6,7].

Compared to fixed-field IMRT, volumetric modulated arc therapy (VMAT) delivers dose in a rotational manner. VMAT seems a logical next step in IMRT delivery, because increasing the number of IMRT beams increases the degrees of freedom [8]. Furthermore, by using dynamic multi-leaf collimator motion, variable dose rate and gantry rotation velocity, VMAT improves dose distribution within the tumor and doses to organs at risk are often reduced and delivery time shortened. For these reasons, rotational arc delivery is often not only marketed as faster delivery, but also for being at least equal, if not superior, for normal tissue sparing in the head and neck or nasopharyngeal tumor treatment in man [9,10].

For stage 4 sinonasal tumors in dogs, and specifically, if a boost dose is added to the gross tumor volume (GTV), it is often difficult to maintain low organ at risk dose (eye, lacrimal glands, rostral brain), while maintaining adequate target dose coverage. IMRT has improved the situation from the 3D-conformal treatments, and the standard for target coverage with IMRT was recently adapted for veterinary medicine [11]. Organ at risk tolerance or the respective dose-volume constraints, however, are not standardized in veterinary medicine. A dose-volume constraint of less than 15 Gy to 60% of the OAR volume (D60<15 Gy) for the ocular bulb has been recommended, when treating nasal tumors with a commonly used protocol of 10x4.2 Gy [5,12]. With the same protocol, a threshold dose to avoid keratoconjunctivitis sicca of a mean dose of less than 20 Gy (D_mean_<20 Gy) was proposed as dose constraint for lacrimal glands [13]. In advanced stage 4 sinonasal tumors, also dose to normal brain could be of concern, since the tumor extends up to the lytic cribriform plate or even shows intracranial extension [14]. To reduce the doses to organs at risk, treatment plans are commonly optimized using point (dose-volume) constraints, aiming for doses as low as reasonably possible.

Currently, it is not known if fixed-field IMRT versus a volumetric arc rotational treatment changes the dose to organs at risk. Clinically, advanced-stage canine sinonasal tumors treated with rotational tomotherapy have yielded only mild ocular and no obvious brain side effects [14]. The latter, however, might not have been detected due to the short life span of the dogs after irradiation of sinonasal tumors. It is likely that side effects will increase if patients are treated with higher doses, as in a boost treatment. At the same time, tumor control probability and therefore survival time increases with dose escalation [7,15,16]. If dogs survive for a prolonged period of time with ameliorated protocols, brain toxicity could be of major concern in the future.

In a pilot series of 9 canine patients with sinonasal tumors (various stages) treated with simultaneously integrated boost, mean ocular doses to 60% of the ocular volume ranged from 4.2 to 23.7 Gy and were higher than the recommended 15 Gy in 7/9 patients and in 10 of 18 eyes [6]. Mean lacrimal gland doses ranged from 1.6 to 35.2 Gy, and hence were higher than the recommended 20 Gy in 3/9 dogs (unpublished data) [6]. While these patients did not have severe acute ocular toxicity, and no late toxicity was observed in the limited observation time, late toxicity could form in the long-term. Long-term toxicity becomes highly relevant specifically in the light of the expected longer tumor control with a higher boost dose.

The aim of our study is to further exploit the advantage of accuracy with intensity modulated radiation by increasing the dose with boost treatments. In order to find the best balance between toxicity to organs at risk and high tumor dose, we investigated if fixed-field IMRT versus rotational volumetric arc treatment technique leads to a relevant dosimetric difference in terms of organs at risk. Our working hypothesis is that VMAT planning yields lower organ at risk doses in eyes, lacrimal glands, corneas, and brain. The study involves re-planning of CT datasets of 10 dogs with advanced, stage 4 sinonasal tumors for both fixed-field IMRT and VMAT. Prescribed dose to the planning target volume (PTV) is a 10x4.2 Gy protocol with a simultaneously-integrated boost (SIB) to the gross tumor volume (GTV) (+20%, 48.3 Gy) and the organ at risk doses will be compared. As a secondary measure, time of delivery and delivered monitor units are compared.

## Materials and methods

### Study aim and design, patient and target volume characteristics

Our main aim was to investigate a possible advantage of VMAT versus fixed-field IMRT in lower organ at risk doses dose in eyes, lacrimal glands, corneas and brain.

This study was a retrospective, analytical, observational design. We applied a theoretical planning approach by including 10 pre-existing computed tomography (CT) datasets from client-owned dogs with CT imaging confirmed stage 4 malignant sinonasal tumors treated with radiation therapy at the Division of Radiation Oncology of the Vetsuisse Faculty, University of Zurich. Age, weight and breed of included dogs were documented. Target volumes (gross tumor volume (GTV), clinical target volume (CTV) and planning target volume (PTV) as well as organs at risk (eyes, corneae, lacrimal glands, and the brain) were delineated in a facility internal standardized manner as previously published by our research group [6]. Absolute target and OAR volumes (cm^3^) and relative boost volumes (%) were documented. Ethical approval was not necessary for this study, as all datasets were from client-owned dogs that had previously undergone regular treatment and data was retrospectively included.

### Treatment planning, dose prescription and constraints for optimization

Each dataset was planned with both, fixed-field IMRT and VMAT by one of two board-certified veterinary radiation oncologists (DACVR (Radiation Oncology)). The plans included a 10-fraction protocol with two different dose levels (simultaneously integrated boost, SIB): 48.3 Gy was prescribed to the GTV and 42 Gy to the PTV [6,11].

For both plans, the dose (to GTV and PTV) was prescribed to the mean (D_50%_) as previously reported [11,17]. For adequate PTV and GTV coverage, also D98/95 had to be fulfilled: 98% of the target volume had to be covered by 95% of the prescribed dose (e.g. 98% of the GTV received ≥45.9 Gy and 98% of the PTV receives ≥39.9 Gy). The near maximum dose (D_2%_ = D_near-max_) was set to D2/115 (e.g. max 2% of GTV as well as the PTV received ≥ 55.5 Gy (115%)). D50, D98, and D2 were documented for each target volume and plan, respectively.

The treatment planning was done using the software Eclipse^™^ Planning system version 15.1.25, including Photon Optimizer with fine settings (1.25mm) (Varian Medical Systems, Palo Alto, CA) with a 6MV linear accelerator (Clinac iX, Varian, Palo Alto, US). The fixed-field IMRT plans had a standard setup of 5 fields (sliding window) with various collimator angles and with bolus placement in dorsal fields if needed for adequate dose build-up at the skin surface. The VMAT plans consisted of one counterclockwise arc with length 359° without bolus placement and one clockwise arc with arc length of 160–200° (from gantry position 260–280° to 80–100°) with bolus placement, with standard 178 control points for a full arc. Collimator angles were set to 30° and 330° for each arc to minimize the tongue and groove effect.

Optimization efforts were based on the dose-volume histograms (DVHs), but included one biology-based parameter for the brain. After adequate target coverage as described above, the highest priority was a generalized equivalent uniform dose (gEUD) of lower than 26.47 Gy for the brain (a = 4), corresponding to a normal tissue complication probability (NTCP) of <5%. For that purpose, the brain was defined as the intracranial volume minus the GTV. Parameter sets from Burman et al. (a = 4, m = 0.15, TD50 = 60 for brain: alpha/beta value = 2) were applied, based on fits to human normal tissue data compiled by Emami et al. as previously described. In order to adjust for fraction size and fraction number in the new 10-fraction protocol, the parameter gEUD was then converted to a biologically equivalent gEUD using the linear-quadratic model [18–20]. Next, we aimed at fulfilling the following constraints for (peri-) ocular organs at risk: dose volume constraint of D60<15 Gy for the ocular bulb [5], dose volume constraint of Dmean<20 Gy for the lacrimal glands [13], dose volume constraint D2<35.4 Gy for the cornea [12,21]. Further minimization to OAR doses, as done in clinical routine, was undertaken, if this was possible without losing target coverage. Target doses D2%, D98%, D50% (dose to 2%, 98%, 50% of the respective target volume), actual absolute values of aspired OAR constraint doses and brain gEUD and NTCP were reported. Whether or not constraints were met were documented per OAR and per dog.

### Monitor units and time of delivery

Monitor units (MU) of all fields (IMRT plans) or arcs (VMAT plans) were summed up to lead to a total MU number per plan. In order to measure the time of delivery, an experienced radiation therapist arranged all fields (IMRT plans) in a logical order (all fields with bolus one after the other, smallest gantry rotation degree in between two individual fields). Time of delivery was then measured in minutes by irradiating all plans in air and measuring the time of beam-on time of the first to the last treatment field or arc.

### Statistical analysis

Data was coded in Excel and analyzed with a commercial statistical software package (IBM^®^ SPSS^®^ Statistics, Version 27 (IBM Corp., Armonk, NY, USA) and RStudio (version 1.4.1103, https://www.r-project.org/). Shapiro–Wilk testing was carried out to assess normality. Mean values were depicted with their standard deviation (SD) and median values with their interquartile range (IQR). As not all variables can be assumed to be normally distributed, related-sample Wilcoxon test was used for paired observations (testing differences of organs at risk doses, monitor units and time of delivery between the plans). Differences were considered significant at p-values <0.05. OAR constraint doses and delivery times and number of MUs were visualized with box-plots.

## Results

### Patient and target volume characteristics

Ten dogs were included into this retrospective analysis and their data are presented in Table 1. Median age of the dogs was 10.3 years (IQR: 3.4, range: 5.6–16.0), median weight 25.9 kg (IQR: 20.7 kg, range: 8.3–60.0 kg). Median gross tumor volume (GTV) was 52.8 cm^3^ (IQR: 78.5 cm^3^, range: 15.3–241.1 cm^3^), median clinical target volume (CTV) was 124.6 cm^3^ (IQR: 100.4 cm^3^, range: 27.4–371.3 cm^3^), median planning target volume (PTV) was 158.2 cm^3^ (IQR: 124.9 cm^3^, range: 41.6–442.5 cm^3^), and median relative boost volume 32.44% (IQR: 15.45%, range 19.7–75.5%).

**Table 1:** Patient characteristics and target volumes.

Patient	Age [years]	Weight [kg]	Breed	GTV [cm^3^]	CTV [cm^3^]	PTV [cm^3^]	Relative boost volume [%]
1	6.0	8.9	Jack Russel Terrier	15.3	27.4	41.6	36.8
2	16.0	11.5	Mixed breed	146.9	176.2	194.6	75.5
3	5.6	37.8	Doberman Pinscher	105.7	175.2	227.8	46.4
4	11.4	34.5	Labrador Retriever	96.7	229.8	304.5	32.7
5	10.2	29.4	Golden Retriever	71.3	163.5	221.6	32.2
6	9.1	60.0	Irish Wolfhound	241.1	371.3	442.5	54.5
7	9.8	26.7	Shar-Pei	34.2	84.6	121.8	28.1
8	10.3	25.0	Collie	23.8	85.7	120.8	19.7
9	13.1	15.7	Spanish Water Dog	21.1	72.6	89.1	23.8
10	13.2	8.3	Cairn Terrier	28.4	69.2	94.9	29.9

CTV: Clinical target volume, GTV: Gross tumor volume, PTV: Planning target volume.

### Treatment planning, organs at risk and constraints

**Fig 1:**
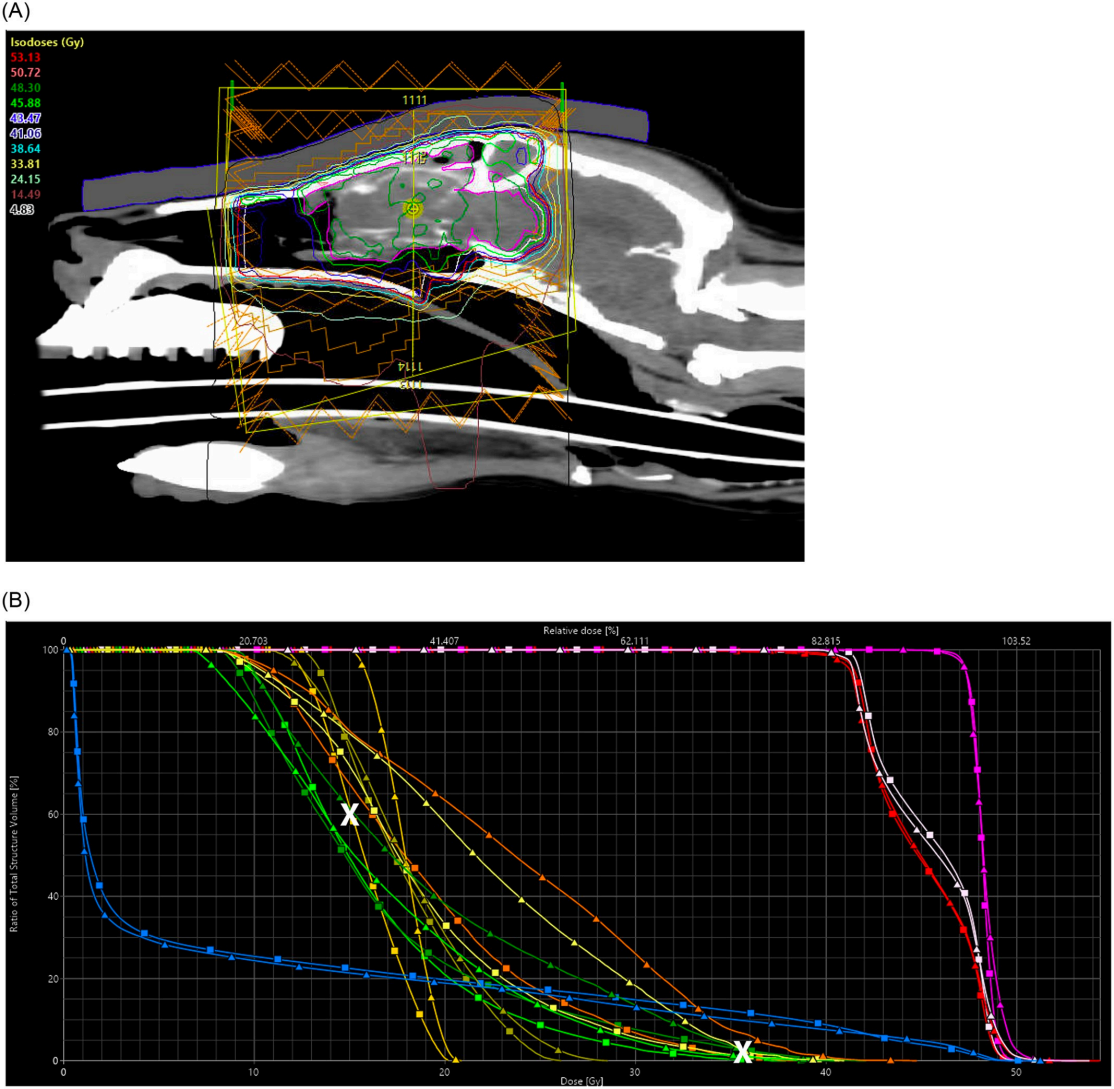
Radiation therapy plan and dose volume histogram. (A) Sagittal computed tomography image of a dog with a stage IV sinonasal tumor with marked intracranial invasion with gross tumor volume (GTV) shown in pink and radiation dose with isodose lines of the IMRT plan in color. (B) Comparison of the dose volume histograms of target volumes (GTV pink, CTV light pink, PTV red) and organs at risk (left cornea orange, right cornea yellow, left eye dark green, right eye light green, lacrimal glands darker yellow, brain blue) between fixed-field IMRT (squares) and VMAT (triangles) plan of the dog depicted in A. The aspired dose volume constraints for eyes (D60<15 Gy) and cornea (D2<35.4 Gy) are depicted with a white X.

Both VMAT and fixed-field IMRT planning resulted in isodose distributions that covered GTV, CTV, and PTV as intended (Table 2 and Fig 1).

**Table 2:** Mean relative doses for target volumes.

Volume	Plan	D2% (mean ± SD) [%]	D50% (mean ± SD) [%]	D98% (mean ± SD) [%]
GTV	IMRT	102.9 ± 1.4	100.3 ± 0.5	96.7 ± 1.1
	VMAT	103.9 ± 2.1	100.4 ± 0.8	96.5 ± 1.1
CTV	IMRT	102.5 ± 1.2	98.7 ± 1.4	85.7 ± 2.3
	VMAT	103.7 ± 2.4	98.9 ± 1.2	86.5 ± 1.2
PTV	IMRT	102.4 ± 1.2	97.1 ± 1.9	84.1 ± 1.4
	VMAT	103.5 ± 2.4	97.0 ± 2.1	84.1 ± 0.8

CTV: Clinical target volume, GTV: Gross tumor volume, IMRT: Intensity modulated radiation therapy, PTV: Planning target volume, VMAT: Volumetric modulated arc therapy.

**Fig 2:**
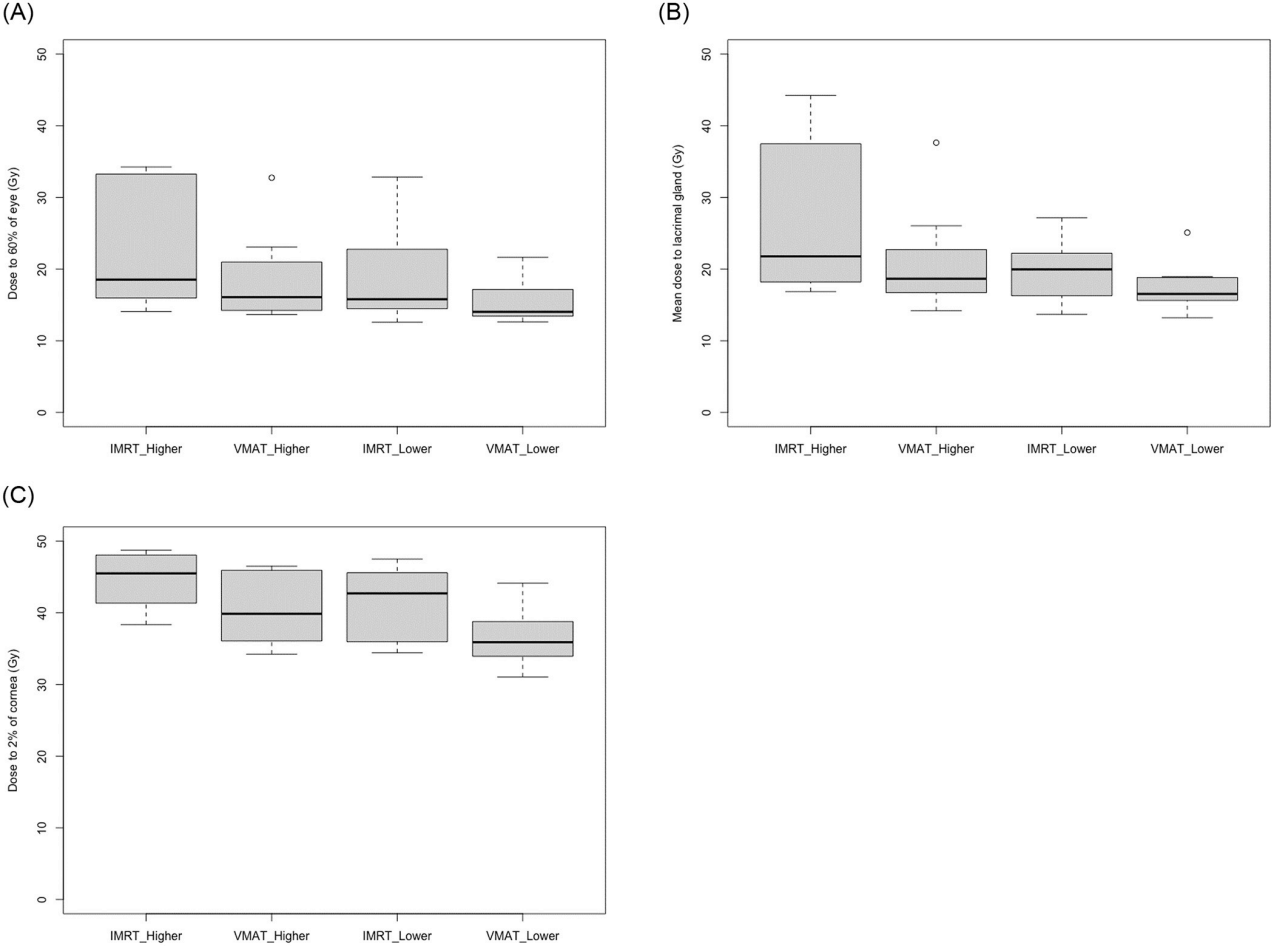
Mean constraint dose to organs at risk. Mean constraint dose to (A) 60% of the eye, (B) the lacrimal glands and (C) the dose to 2% of the corneae in the respective organ at risk in the high and lower dose region for each IMRT and VMAT treatment plans, respectively (for individual P-values see Table 3). IMRT: Intensity modulated radiation therapy; VMAT: Volumetric modulated arc therapy.

Median NTCP for brain was <5% as required and was not different between fixed-field IMRT and VMAT plans (p = 0.29). Mean dose for all other constraints was significantly lower in VMAT compared to fixed-field IMRT plans (Table 3 and Fig 2). Constraints were met in 21/40 eyes, 7/40 corneae, and 24/40 lacrimal glands in total. When evaluating if constraints were met per dog, this was the case in 8/20, 1/20 and 10/20 dogs for both eyes, corneae and lacrimal glands, respectively. Constraints were met in 3/10 eyes in the higher dose area in IMRT and 5/10 VMAT plans, respectively, and 6/10 eyes in the lower dose area in IMRT and 7/10 VMAT plans, respectively. Constraints were met in the lacrimal gland in the higher dose area in 4/10 IMRT and 6/10 VMAT plans and in the lower dose area in 5/10 IMRT and 9/10 VMAT plans, respectively. For the cornea in the high dose area, constraints were met in 0/10 IMRT and 1/10 VMAT plans, respectively. For the cornea in the low dose area, constraints were met in 2/10 IMRT and 4/10 VMAT plans, respectively.

**Table 3:** Organ at risk volume and actual constraint dose per plan.

OAR constraint	OAR Volume (median, IQR) [cm^3^]	IMRT plan (median, IQR) [Gy]	VMAT plan (median, IQR) [Gy]	P-value of plan comparison
Eye_higher_ D60% *[aspired constraint 15 Gy]*	5.5, 0.7	18.5, 17.6	16.1,7.4	<0.01
Eye_lower_ D60% *[aspired constraint 15 Gy]*	5.4, 1.0	15.8, 9.6	15.1, 4.2	<0.05
Lacrimal gland_higher_ Dmean *[aspired constraint 20 Gy]*	0.1, 0	21.8, 20.5	18.6, 7.0	0.01
Lacrimal gland_lower_ Dmean *[aspired constraint 20 Gy]*	0.1, 0	20.0, 6.3	16.5, 3.2	<0.05
Cornea_higher_ D2% *[aspired constraint 35*.*4 Gy]*	1.9, 1.2	45.5, 6.8	39.9, 10.0	<0.01
Cornea_lower_ D2% *[aspired constraint 35*.*4 Gy]*	2.2, 0.8	42.7, 9.0	35.9, 6.2	<0.01
Brain gEUD *[aspired constraint 26*.*4 Gy]*	81.3, 24.1	25.8, 1.7	25.3, 2.9	0.24
NTCP Brain-GTV *[required constraint <5%]*		3.3%, 2.1	3.1%, 3.3	0.29

D2%, D60%: Dose to 2% or 60% of the volume, Eye_higher_: Eye in the higher dose region, gEUD: Generalized equivalent uniform dose, IMRT: Intensity-modulated radiation therapy, OAR: Organs at risk, NTCP: Normal tissue complication probability, VMAT: Volumetric modulated arc therapy.

### Monitor units and time of delivery

The mean total monitor units (MU) was 2446.0 (±1015.5) for fixed-field IMRT plans and 1033.2 (±107.8) for VMAT plans, respectively. There was a significantly higher total MU number in IMRT plans compared to VMAT plans (p<0.01).

**Fig 3:**
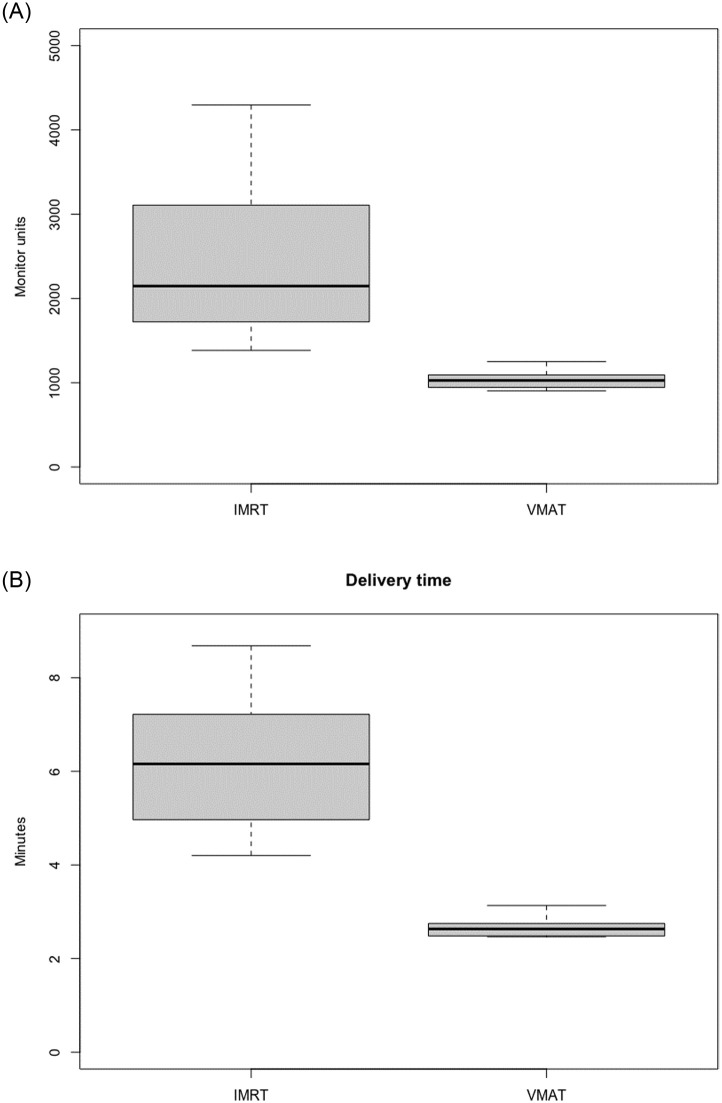
Mean monitor units and delivery time. Monitor units and delivery time of fixed-field IMRT versus VMAT plans: (A) The mean number of monitor units was significantly higher for fixed-field IMRT (2446.0 (±1015.5)) versus VMAT plans (1033.2 (±107.8)) (p<0.01). (B) The median delivery time for fixed-field IMRT plans (6.16 minutes, IQR 2.2) was significantly longer compared to VMAT plans (2.63 minutes, IQR 0.25) (p<0.01). IMRT: Intensity modulated radiation therapy; VMAT: Volumetric modulated arc therapy.

The median delivery time was 6.16 minutes (IQR 2.2, range 4.2–8.68 minutes) for fixed-field IMRT plans and 2.63 minutes (IQR 0.25, range 2.47–3.13 minutes) for VMAT plans (Fig 3). Delivery time for fixed-field IMRT plans was significantly longer than for VMAT plans (p<0.01) (Fig 3).

## Discussion

This study presents a comparison of volumetric intensity modulated arc therapy (RapidArc) with fixed-field IMRT for the treatment of dogs with stage 4 sinonasal tumors with a simultaneously-integrated boost protocol. Clinically acceptable plans were achieved with both, VMAT and fixed-field IMRT. As hypothesized, VMAT plans yielded significantly lower organ at risk doses in eyes, corneae, and lacrimal glands. Also, treatment times were significantly shorter and the number of monitor units to deliver the dose with VMAT were significantly lower.

VMAT is a recent planning and treatment approach in veterinary medicine and only a few studies exist in dogs [5,14,22–24]. VMAT is associated with faster delivery times than IMRT, but more time is needed for quality assurance/ plan verification compared to 3DCRT and—depending on computer performance—treatment planning [9,10,25]. IMRT has led to marked sparing of ocular OARs in dogs [5]. Direct comparisons of IMRT and VMAT plans have not yet been published, as far as we know.

Due to the advanced stage of the disease of the cases chosen for our study and the prioritized constraint of dose to the brain, however, not all of the aspired (peri-) ocular dose constraints could be met for this boost-protocol. Even with highly conformal techniques, radiation fields used to treat the upper respiratory track (nasosinus, nasopharynx) often include a portion of the brain [26]. The relative boost volumes of our study participants was large, with about 1/3 of the PTV, and there was marked intracranial extension in most cases. Limiting the dose to the brain to an NTCP of <5% (represented by a gEUD <26.47 Gy during the optimization process) was a clinical decision, based on balancing of curative benefits and the risk of potentially fatal consequences for a toxicity such as brain necrosis for the patient. An earlier study, investigating boost radiation therapy in sinonasal tumors in dogs, reported severe brain toxicity in 22% (4/18) of the dogs with computer-based Cobalt-60 plans with 2–3 fields [27]. Radiation response in the dog brain was studied in the past, but mostly with high doses to a large portion of the brain [28–30]. Radiation dose-volume effects and normal tissue complication probability in the brain of dogs, however, have not been specifically collected and evaluated to date, hence the analysis is based on assumptions from human data including the dog’s different brain volume and the protocol’s different fraction size [18,31–34]. Similarly, dose constraints to (peri-) ocular organs are rarely described in veterinary radiation therapy [12]. Lawrence et al. (2010) described no clinically relevant toxicity to the eye with the constraints D60<15 Gy in 31 dogs treated with IMRT and a 10x4.2 Gy protocol, compared to an historical control with mean doses of 33.6 Gy to eyes and 64% severe late toxicity with loss of vision in 20/36 dogs (unilateral blindness n = 13, bilateral n = 7) with Cobalt-60 treatment plans [5]. While Lawrence et al. were able to limit the dose to the eye to D60<15 Gy in IMRT patients, it is possible that ocular dose could be higher without causing severe toxicity. They included both nasal cavities into the PTV (regardless of whether the GTV was uni- or bilateral) and therefore did not see a difference between the mean doses to the two eyes in 25/27 of the evaluated dogs. In contrast, a pilot study of 9 dogs treated with this boost-protocol, ocular doses were higher than the recommended 15 Gy in 7/9 patients and in 10 of 18 eyes, with one of the eyes often being in the high and one in the lower dose area. Although late effects were not a primary outcome of the mentioned pilot study, 7/9 dogs were followed longer than 6 months (mean 280 days) and did not develop clinically relevant ocular late toxicity [6]. The same lack of toxicity might hold true for the constraints of the cornea and lacrimal gland. Lacrimal gland toxicity was examined in 15 dogs treated with 10x4.2 Gy and did not result in keratoconjunctivitis sicca (KCS) if mean dose to the lacrimal gland was <20 Gy, in contrast to 5/7 lacrimal glands with dose >20 Gy. Mean dose was also significantly lower in eyes that did not develop KCS (mean 11.6 Gy) versus eyes that developed KCS later on (mean 30.8 Gy) [13]. Since ocular toxicity up to (unacceptable) loss of an eye was described in earlier studies and since this could have been due to KCS and/or keratitis, we decided to add a separate constraint for the cornea [21]. Sparing the whole ocular bulb with the constraint described above might still allow excessive dose to the cornea. With a corneal constraint, (maximum) dose deposition can be guided more carefully away from the cornea. Dose to the cornea was rather high with near-maximum (D2%) dose of 45.5 Gy and 39.9 Gy for the eye_higher_ for IMRT and VMAT plans, respectively and the aspired constraint of 35.4 Gy was only met in 7 of 40 eyes. While the study of Soukup et al. did not describe dose to the cornea, mild peripheral corneal pigmentation was reported after RT in 6 dogs, this remained stable or decreased at a later time point [6]. To the best of the authors’ knowledge, no corneal tolerance information exists in the sparse veterinary literature [12]. It was therefore necessary to adapt tolerance doses known from human patients by recalculating these to match the applied fractionation schedule [21].

In planning studies of nasal or head and neck tumor irradiation in humans, volumetric intensity-modulated arc (VMAT, RapidArc) was superior over conventional fixed-field IMRT in terms of higher PTV homogeneity with double arc plans [9], lower MUs of up to 78% [9,10] and shorter delivery times. Overall, RapidArc plans provided at least similar sparing of organs at risk according to a previous study [9]. Speed of delivery is an additional advantage of RapidArc as it reduces the risk of intrafraction movements, decreases time of anesthesia and allows the treatment of more patients in a shorter time. Lower monitor units proportionally reduce the dose to healthy organs distant to the PTV, dose arising largely from collimator transmission and scatter radiation from the linear accelerator [35,36]. In our study, the number of MUs was decreased by 58% and the delivery time shortened by 57% for VMAT plans.

Volumetric arc techniques have been found to produce dosimetrically comparable plans, with often lower doses to organs at risk also for anatomically simpler sites such as prostate and benign intracranial cancers in humans [37,38]. In humans as well as in dogs, nasopharyngeal tumors are considered to be more complex, due to the shape and the anatomical crowding of sensitive organs around the sinonasal cavity [2,10]. Planning for concave tumor shapes, such as tumors with orbital extension—in very close proximity or even wrapping around an eye—usually profits from increasing the number of treatment field angles added [2].

We acknowledge limitations of this rather small treatment planning approach presented herein, which could direct future research. The dogs included varied in weight and could therefore also vary in nasal cavity size. All dogs had advanced stage 4 nasal tumor, which by definition (cribriform plate between nasal cavity and brain destroyed) is a tumor in direct contact (or even invasion into) the brain and close proximity to ocular structures. While we chose brain as most important OAR with a fixed NTCP upper limit allowed, one could argue that dogs with sinonasal tumors often succumb to their disease after around 1–1.5 years and might therefore not live long enough to exhibit late toxicity to the brain [5,39–43]. Brain toxicity within a few months after radiation therapy has mostly been reported in dogs with more toxic, severely hypofractionated/ stereotactic protocols [44]. Severe ocular toxicity, on the other hand, can have a dramatic impact on quality of life of a pet dog and should therefore be avoided at all cost. Given that any tumor control probability depends—at least in part—on the extent of disease or tumor size (e.g., number of tumor cells to be killed), some dogs with stage 4 sinonasal disease might not tolerate a dose-escalated, boosted protocol with an acceptable risk of late toxicity to OARs. Especially in tumors centered around the junction of nasal cavity and frontal sinus to brain, high doses to eyes and/or brain might not be avoidable. In such a disease setting, the planner will have to decide to a) compromise on some of the organ at risk constraints, in order to provide adequate dose coverage to the target or b) not treat the patient with this protocol. Compromising target dose is non-negotiable, in order to ensure an expected tumor control probability. Clinicians, however, sometimes intentionally allow some underdosages to the target volumes at critical sites (e.g., close to OARs), in spite of an intended homogenous dose protocol. However, these underdosages in fear of side effects are often based on intuition and rarely follow evidence-based rules. We see this specifically in veterinary medicine, where dosimetric constraints to organs at risk have not been carefully collected and summarized. Such an unstandardized course of action, however, can endanger the patient’s tumor control in an unpredictable way and renders outcome studies difficult to interpret. The OAR constraints applied herein can be used as a guideline, but are based on weak dog data (eyes, lacrimal glands) or human data (brain, cornea) and might be overly conservative. Only adequate target volume coverage and upper brain NTCP limit were required in our study, while other factors, such as monitor units, were not limited. This led to high MU numbers in fixed-field IMRT plans and most likely to the wide range of MUs and delivery times. IMRT plans can be performed using multiple fields and beam angles. While adding more fields (such as a 9- or 12-field approach) to our IMRT plans would most likely have led to a more conformal plan and better sparing of organ at risk, this would have led to longer treatment times and was therefore omitted.

## Conclusions

In conclusion, our findings suggest that dogs with advanced-stage sinonasal tumors can profit from a lower dose to OARs from VMAT treatment, when a 20% SIB-enhanced radiation protocol is to be delivered, as well as from lower MUs and faster delivery time when using our set-up. While we were not able to reach all of the constraints with either of the two planning techniques, more constraints were met with VMAT. This suggests that VMAT should be preferred over our regular 5-field IMRT approach. Currently it remains unclear, however, which dose to OARs can be considered safe—without causing excessive discomfort to the patient or even loss of functionality—in dogs irradiated for sinonasal tumors.
